# Exosomal microRNA-Based therapies for skin diseases

**DOI:** 10.1016/j.reth.2023.12.005

**Published:** 2023-12-23

**Authors:** Chen Jibing, Liang Weiping, Yang Yuwei, Feng Bingzheng, Xu Zhiran

**Affiliations:** Ruikang Hospital Affiliated to Guangxi University of Chinese Medicine, Nanning, Guangxi, China

**Keywords:** Exosome, microRNA, Mesenchymal stem cell, Skin disease, Skin aging

## Abstract

Based on engineered cell/exosome technology and various skin-related animal models, exosomal microRNA (miRNA)-based therapies derived from natural exosomes have shown good therapeutic effects on nine skin diseases, including full-thickness skin defects, diabetic ulcers, skin burns, hypertrophic scars, psoriasis, systemic sclerosis, atopic dermatitis, skin aging, and hair loss. Comparative experimental research showed that the therapeutic effect of miRNA-overexpressing exosomes was better than that of their natural exosomes. Using a dual-luciferase reporter assay, the targets of all therapeutic miRNAs in skin cells have been screened and confirmed. For these nine types of skin diseases, a total of 11 animal models and 21 exosomal miRNA-based therapies have been developed. This review provides a detailed description of the animal models, miRNA therapies, disease evaluation indicators, and treatment results of exosomal miRNA therapies, with the aim of providing a reference and guidance for future clinical trials. There is currently no literature on the merits or drawbacks of miRNA therapies compared with standard treatments.

## 1 Introduction

### 1.1 MicroRNA therapy for diseases

Functional ribonucleic acids (RNAs) include both coding and noncoding subclasses. MicroRNAs (miRNAs) are small and highly conserved, noncoding, single-stranded RNA, containing 19–25 nucleotides. One mature miRNA generally targets several to dozens of messenger RNAs (mRNAs), binding to the 3′-untranslated region and resulting in the translation-suppression or degradation of the target mRNAs [1]. In humans, two mature miRNAs, miR-3p and miR-5p, are derived from 3′ and 5′ ends of premature miRNA, respectively. miRNAs participate in cell development, proliferation, and apoptosis, and an endogenous or exogenous miRNA might exert different effects in different cell types of the same species, and might exhibit similar effects in the same cell type of different species [2].

Skin is the largest organ of the human body, and a large amount of highly regulated miRNAs are required for its development and function. miRNAs derived from skin cells are involved in skin diseases, wound healing [3], cell apoptosis, aging, and pigmentation [4]. The different expression levels of miRNAs between sick and healthy skin cells (e.g., fibroblasts, macrophages, endotheliocytes, and keratinocytes) indicate that those miRNAs whose expression decreases significantly in skin lesions have the potential to become drugs to treat skin diseases. Fortunately, these drugs can be produced in the laboratory as miRNA mimics, agomirs, inhibitors, and antagomirs. An miRNA agomir (or antagomir) is a specially labeled and chemically modified miRNA mimic (or inhibitor), with higher affinity for cell membranes and higher stability and inhibitory effects in *in vivo* experiments. The endogenous miRNAs that are decreased in diseases can be supplied using exogenous miRNA mimics or miRNA agomirs. In contrast, miRNA inhibitors or miRNA antagomirs can be supplied to inhibit excessively-expressed miRNAs in skin lesions [5].

In recent years, many scholars have attempted to apply miRNA-based exosome therapies to skin diseases. These miRNA-based therapies have reported significant efficacy in various preclinical studies, and the corresponding clinical studies are at different stages of research. The adverse events of miRNAs are referred to as the “too many targets for miRNA effect”, which are inevitable because of the special complementary nucleotides between miRNAs and their targets [6]. At present, a phase I clinical study using miRNA therapy to treat skin wounds has been completed, showing clear therapeutic effects [7,8]. This review focuses on the effectiveness of preclinical studies to treat skin diseases, including full-thickness skin defects, diabetic ulcers, psoriasis, skin burns, systemic sclerosis, photoaging, and hair loss.

### 1.2 Ideal delivery modes of miRNAs

miRNAs are usually protected by exosomes to avoid RNase degradation in the extracellular fluid [9]. Exosomes are extracellular vesicles of 30–150 nm, with the same lipid bilayer structure as their parental cells, which can cross various physiological barriers in the body and transmit information between cells. Therefore, the miRNAs carried by exosomes can penetrate neighboring cells and control the translation of their target mRNAs [10]. Mesenchymal stem cell (MSC)-derived exosomes (MSC-Exos) currently the most widely used type of exosomes, being suitable for local administration, with low immunogenicity [11]. Furthermore, MSC-Exos have similar functional elements to their parent MSCs, which can naturally target inflammatory sites and readily fuse with inflammatory cells [12], apoptotic cells, the vascular endothelium, and tissue-derived stem cells, to modulate their behavior [13]. Notably, owing to their nanosize and biological origin, MSC-Exos possess outstanding capabilities to penetrate the stratum corneum barrier and reach the deep epidermis, dermis, and subcutaneous tissue [14]. When studying the function of exosomes, the exosome inhibitor GW4869 is often used to inhibit the release of exosomes by MSCs and thereby reverse the therapeutic effect [15].

Although there are many types of active ingredients in exosomes, their content is usually low. The techniques to increase the abundance of miRNAs in exosomes include both viral and nonviral methods [16]. Most current applications of exosomal miRNA therapy for skin disease treatment use lentiviral vector-mediated gene delivery. MSCs are easily transfected by lentiviruses, which is more efficient than nonviral methods and ensures stable and long-term transcription of miRNAs [17]. Meanwhile, lentiviral vectors show better safety after insertion into the MSC genome than other virus types [18]. Lentivirus transfection is highly efficient for miRNA expression; however, the production costs are high and they are associated with adverse immune responses [19]. In contrast, nonviral transfection is easy to operate, with low immunogenicity, and the common types are electroporation and liposomes [20]. The electroporation method uses a high voltage electric pulse to reversibly perforate the cell membrane and introduce the miRNAs into the cytoplasm or nucleus [21]. Liposomes are nanoscale vesicles with a structure similar to cell membranes, which encapsulate miRNAs and fuse with MSCs [22] or MSC-Exos [23] to introduce miRNAs into the target cells. Exosomes with an overabundance of, or depleted for, certain miRNAs are usually abbreviated as “Exo-miR”, “miR agomir”, “Exo-miR inhibitor” or “miR antagomir”.

### 1.3 Skin histology across species

The skin is the body's largest organ in various vertebrates. It serves as a barrier and immune defense against pathogens. After skin injury, a cascade of events, including inflammation, granulation tissue formation, amplification, and differentiation of basal layer stem cells, contributes to wound repair. Skin-resident and recruited immunocytes work together with inherent skin cells to clear invading pathogens and debris, and restore skin homeostasis. Human skin consists of the epidermis, dermis, and subcutaneous tissue, in which skin appendages, including nails, hair, sweat glands, and sebaceous, are distributed. The epidermis is composed of a stratified squamous epithelium, in which keratinocytes are the main cells, accounting for about 95 %. Other cells include melanocytes, inflammatory cells, and macrophages. There is a thin fibrous layer between the epidermis and the dermis, called the basement membrane, in which skin stem cells are located, playing a central role in the process of physiological remodeling or repair. The dermis is located deep in the epidermis and is composed of connective tissue, which can cushion the pressure on the body. The dermis contains an extracellular matrix composed of collagen fibers and elastic fibers, which is embedded in hyaluronic acid and proteoglycan to provide skin tension and elasticity. The blood vessels in the dermis provide nutrients for the cells in the dermis and epidermis, and also remove waste generated after metabolism. Once the skin is damaged, if the wound is mild, the body can produce collagen and fibrin on its own to repair the wound. Scars can form after healing of severely injured skin, and sometimes the color of the skin changes. The thickness of animal and human skin varies depending on the location, for example, the skin below the human eyes and around the eyelids is the thinnest (about 0.5 mm), and is the site of the earliest occurrence of skin aging, such as “crow's feet” or other wrinkles. The human skin on the palms and soles of the feet is the thickest, with a thickness of approximately 4 mm.

The skin of rodents contains the epidermis and dermis, and the interphase epidermis and dermis is flat, whereas in humans it is highly undulated [24]. In addition, the rodent skin dorsum is covered with dense hair, and the rodent the hair cycle is usually three weeks, whereas human hair cycles usually last many years [25]. Additionally, mouse skin has a distinct panniculus carnosus, which is a thin skeletal muscle layer found only at the platysma in humans [26]. Compared with that of humans, the skin of rats is loose, with poor elasticity and a lack of strong adherence to the underlying structures [27]. This property of rat skin plays a significant role in wound healing. Thus, the research results obtained using rodent models might not be perfectly replicated in human skin.

## 2 Skin diseases

### 2.1 Full-thickness skin defects

Skin wound healing is a complex process that involves multiple highly coordinated steps, including inhibiting the inflammatory response, promoting epithelial formation and wound contraction by fibroblasts, collagen deposition, and remodeling. Trauma, surgery, and acute or chronic diseases can all lead to full-thickness skin defects, characterized by scar formation, altered pigmentation, and persistent ulcers in clinical practice [28]. Failure to heal within three months can be considered as a difficult-to-heal wound. The current clinical therapies include physiotherapy, biological dressing, application of recombinant human epidermal growth factor, and tissue engineering skin transplantation [29]. The purposes of miRNA therapy for full-thickness skin defects include promoting angiogenesis of the dermis, promoting the differentiation of skin stem cells into epidermal cells and fibroblasts, promoting the migration of epidermal cells and fibroblasts to the wound, inhibiting the apoptosis of mature epidermal cells and fibroblasts, and inhibiting skin inflammation. A full-thickness skin defect model [30] is constructed using a knife or biopsy punch to create multiple circular defects after shaving the back of white rats or mice.

#### 2.1.1 miR-19b

miR-19b was previously reported to reduce extracellular matrix degradation and inflammatory damage, while promoting wound healing [31]. Cao et al. [32] assessed H_2_O_2_-treated keratinocytes and found that miR-19b expression decreased as the concentration of H_2_O_2_ increased. In a Transwell co-culture system, adipose-derived stem cell (ADSC) and ADSC-Exo treatment increased the expression of miR-19b in keratinocytes, while GW4869 reduced miR-19b expression, which indicated that the ADSCs shuttled exosomal miR-19b to keratinocytes. Using a dual-luciferase reporter assay, miR-19b in keratinocytes targeted mRNA encoding chemokine CC motif ligand 1 (CCL1), which can increase chemotactic inflammatory cells and enhance wound inflammation. Subsequently, 15 mice were randomly and evenly divided into 3 groups: The control group (phosphate buffered saline [PBS]), the ADSC-Exo/GW4869 group, and the ADSC-Exo group). The two groups of mice subcutaneously injected with exosomes exhibited faster wound healing than the control group, and ADSC-Exos were more effective than ADSC-Exo/GW4869. At day 8, the exosome-treated wounds had thicker granulation tissues and less inflammatory infiltration, with the ADSC-Exo group having the least pathological degree. After ADSC-Exo or ADSC-Exo/GW4869 treatment, the expression of miR-19b and transforming growth factor (TGF)-β increased, and the pathological degree of the ADSC-Exo group was the highest. This study indicated that exosomal miR-19b derived from ADSCs regulates the TGF-β pathway by targeting *Ccl1* mRNA in keratinocytes, thereby promoting the healing of skin defects.

#### 2.1.2 miR-125b

Zhang et al. [33] verified the effect of normoxic or hypoxic exosomes from human umbilical cord-derived mesenchymal stem cells (UCMSCs) on wound healing. Fifteen mice were randomly and evenly divided into three groups: The control group (100 μL of PBS), the normoxic MSC-Exo group (200 μg), and the hypoxic MSC-Exo group (200 μg). At day 3 and 8 after subcutaneous injection, the wound areas in two MSC-Exo groups were both smaller than that of the control group, while there was no significant difference between the MSC-Exo groups. The MSC-Exo groups had significantly more Ki67^+^ cells and fewer terminal deoxynulceotidyl transferase nick-end-labeling (TUNEL)^+^ cells than the control group, which was more obvious in the hypoxic MSC-Exo group, suggesting a better effect of hypoxic MSC-Exos. The level of miR-125b in the hypoxic MSC-Exos was much higher than that in the normoxic MSC-Exos, and similar results were obtained from endotheliocyte absorption experiments. In endotheliocytes, dual-luciferase reporter assays confirmed the direct binding of miR-125b to the mRNA encoding tumor protein p53 inducible nuclear protein 1, which can promote apoptosis of endotheliocytes. The function of miR-125b was further determined by injecting miR-125b agomir or agomir-negative control (NC) into the areas adjacent to skin defect: the wound in the miR-125b agomir group closed more rapidly, with more Ki67^+^ cells and fewer TUNEL^+^ cells. The above results confirmed the angiogenesis promoting effect of miR-125b-mediated wound repair.

#### 2.1.3 miR-126-3p

Ma et al. [30] found that miR-126-3p directly targets the mRNA encoding phosphoinositide-3-kinase (PI3K) regulatory subunit 2 in skin fibroblasts and vascular endotheliocytes. They transfected an miR-126-3p inhibitor into human ADSCs using liposomes, and Exo-miR-126-3p inhibitors were isolated from the culture supernatant after 24 h. Forty-five rats were randomly and evenly divided into three groups: The control group (PBS), the Exo-miR-126-3p inhibitor group (0.2 mL) and the ADSC-Exo group (0.2 mL). After injection in four wound sides, ADSC-Exos partially healed the skin defects at week 4 and completely at week 5, while the skin defects in the Exo-miR-126-3p inhibitor group and control group were only healed partially at week 5. A neat skin structure, reduced infiltration of inflammatory cells, and bulky-organized collagen deposition were observed only in the ADSC-Exo group. The ADSC-Exo group had more new vessels than the miR-126-3p inhibitor group and control group. The above results showed that miR-126-3p could increase collagen deposition and angiogenesis simultaneously in skin defects.

#### 2.1.4 miR-135a

Gao et al. [34] found that miR-135a directly targets the mRNA encoding large tumor suppressor factor 2 (LATS2) in fibroblasts, thereby promoting local fibroblast migration to wounds. They transfected an miR-135a plasmid into human amniotic mesenchymal stem cells (AMSCs) using liposomes, and Exo-miR-135a were isolated from the culture supernatant at 48 h. Twenty-five rats were randomly and evenly divided into five groups: The control group (saline), the 293 T cells-Exo group, the AMSC-Exo group, the Exo-miR-135a group, and the miR-135a antagomir group. Exosomes were mixed with collagen-I (0.5 mL), uniformly coated on the wound surface, and then covered with Vaseline gauze. After 5 days of treatment, the Exo-miR-135a group showed the fastest wound healing compared with the other four groups, and the miR-135a antagomir group showed the slowest wound healing. At day 15, only the wounds in the Exo-miR-135a group had healed completely, with a neat dermis layer, new granulation tissue, and almost no infiltration of inflammatory cells. The above results showed that miR-135a could accelerate wound healing and epithelialization by promoting the migration of fibroblasts.

#### 2.1.5 miR-146a

In lipopolysaccharide-stimulated endotheliocytes, miR-146a [35] was reported to target the mRNA encoding caspase recruitment domain protein 10 (CARD10) and regulate angiogenesis. Chen et al. [36] extracted exosomes from miR-146a-modified ADSCs and explored their therapeutic effect on chronic wound healing. miR-146a mimic and miR-NC were transfected into human ADSCs using liposomes, and Exo-miR-146a and Exo-miR-NC were extracted at 24 h after successful transfection. Ten rats were randomly divided into three groups: The control group (n = 4), the Exo-miR-NC group (n = 3, 1.25 μg/μL in 80 μL), and the Exo-miR-146a group (n = 3, 0.95 μg/μL in 100 μL). The exosomes were injected into four different sites around the skin defects. The scar diameter of the skin defect was measured on day 3, 7, and 11. The scar healing speed in the Exo-miR-146a group was significantly higher than that in the control group and the Exo-miR-NC group before day 7. Compared with the control and Exo-miR-NC groups, the expression of CD31 in the Exo-miR-146a group was significantly increased, which indicated that miR-146a promoted the neovascularization of full-thickness skin defects.

#### 2.1.6 miR-542-3p

In research into antioxidant drugs, miR-542-3p [37] was reported to target the mRNA encoding angiopoietin-2 and promote the angiogenesis of endotheliocytes. Xiong et al. [38] found that the expression of miR-542-3p in the damaged skin of mice was significantly lower than that in normal skin; therefore, they used miR-542-3p to promote wound healing. Bone marrow mesenchymal stem cell (BMSC)-derived exosomes (BMSC-Exos) were isolated using ultracentrifugation, and then loaded with miRNA-542-3p by electroporation. Twenty-seven mice were randomly and evenly divided into three groups: The control group (PBS), the Exo-miR-542-3p group (200 μg), and the BMSC-Exo group (200 μg). Eight days after injection around the wound bed, the wounds in the Exo-miR-542-3p group were smaller than those in the BMSC-Exo group, with narrower edges, and significantly increased re-epithelialization, collagen deposition, and capillary density. The above results showed that miR-542-3p promotes collagen deposition and blood vessel formation in the processes of wound repair.

### 2.2 Diabetic ulcers

Chronic ulcers are one of the most debilitating complications of diabetes mellitus, occurring in about 6 % of all patients [39]. In chronic ulcers, persistent and intense inflammation throughout the body, microcirculation disorders, and the presence of necrotic tissue, combine to prolong the healing process of ulcers [40]. Currently, the treatment of diabetic ulcers includes blood glucose control, hyaluronic acid dressing, surgical debridement, negative pressure bandaging, antibiotics, and hyperbaric oxygen treatment [41]. The above therapies only treat the symptoms and skin ulcers cannot be repaired through the normal wound healing process, and often develop into gangrenous infections, leading to amputation [42]. Appropriate angiogenesis, which supplies nutrients/circulating stem cells and removes waste products, is essential to heal diabetic ulcers [43]. Rodent models of diabetes include the streptozotocin-induced model and the spontaneous model [44]. A single intraperitoneal injection of streptozotocin (100–200 mg/kg in mice and 35–65 mg/kg in rats) can induce diabetes in 2 weeks (>16.8 mmol/L in mice and >20 mmol/L in rats). BKS db/db mice (leptin receptor mutation) are a spontaneous type 2 diabetes model, whose blood glucose starts to rise at 1–2 weeks of age, and can produce a recognizable obesity phenotype at 3–4 weeks of age, with weight loss and polyuria symptoms [45]. Diabetic BKS db/db mice suffer from spontaneous peripheral neuritis, and show significantly slower wound healing.

#### 2.2.1 miR-126-3p

Transplantation of MSCs transfected with miR-126 has been shown to improve angiogenesis in the vascular endotheliocytes of ischemic diseases through the phosphoinositide-3-kinase (PI3K)/protein kinase B (AKT) and mitogen-activated protein kinase (MAPK)/extracellular signal-regulated kinase (ERK) pathways [46]. Tao et al. [47] reported the therapeutic effect of miR126-3p on diabetic ulcers in rats. Human synovium-derived MSCs (SMSCs) were isolated from biopsies of synovial membranes, and then incubated with an miR-126-3p lentiviral vector for 24 h. SMSCs were selected using puromycin, and Exo-miR-126-3p were harvested from the supernatant. Eighteen streptozotocin-induced diabetic rats were randomly and evenly divided into three groups after the wounds were created. Two treatment groups were covered with chitosan hydrogel (CS)-containing Exo-miR-126-3p and CS alone, respectively. Although the wounds in all three groups improved, the sizes in CS group and CS-Exo-miR-126-3p group were both significantly smaller than those of the control group (untreated) at 14 days. Compared with the CS group and control group, the CS-Exo-miR-126-3p group showed a much higher number of blood vessels, enhanced re-epithelialization, thicker granulation tissue, and improved collagen deposition, mature hair follicles, and sebaceous glands. The above results suggested that miR-126-3p could accelerate epithelial regeneration, activate angiogenesis, and promote collagen maturation, demonstrating good potential for treating diabetic ulcers.

#### 2.2.2 miR-132

During an investigation of endotheliocytes, miR-132 [48] was found to target the TGF-β, PI3K/AKT, and Hippo pathways, thereby promoting proliferation and migration. Ge et al. [49] applied this discovery to treat diabetic ulcers in mice. A lentivirus carrying miR-132 was transfected into mouse ADSCs, then Exo-miR-132 were harvested from the supernatant. Thirty streptozotocin-induced diabetic mice were randomly and evenly divided into three groups: The control group (saline), the Exo-miR-132 group (2 μg/μL), and the ADSC-Exo group (2 μg/μL). The flaps were used to cover the full-thickness skin defects to observe the healing status of the wound. On day 0, 3, and 7, 100 μL of saline or exosomes were injected subcutaneously in four points around the wound. At different time points, the control group had the largest area of necrotic flap, and treatment with Exo-miR-132 had a better effect than that of ADSC-Exos, indicating that miR-132 significantly improved the healing of diabetes ulcers. The two exosome groups showed lower levels of edema and subcutaneous venous congestion than the control group, with the most significant effect in the Exo-miR-132 group. In the necrosis and surviving tissue junction, the two exosome groups showed more intensive microvessels and tighter collagen fibers than the control group, with the most significant effect in the Exo-miR-132 group. All these results confirmed that Exo-miR-132 could accelerate the healing of diabetic ulcers by promoting the formation of microvessels.

#### 2.2.3 miR-146a

miR-146a has been found to have two effects, one is binding to the mRNA encoding IL-1R-associated kinase 1 in macrophages, which indirectly inhibits TNFR-associated factor 6 and nuclear factor kappa B (NF-κB), ultimately inhibiting the production of inflammatory cytokines [50]. The other is binding to the mRNA encoding small mothers against decapentaplegic (Smad)4 in keratinocytes, which indirectly promotes vascular endothelial growth factor (VEGF) expression and ultimately enhances vascular regeneration [51]. Li et al. [52] screened out the silk fibroin binding peptide-Gluc-MS2 (SGM) from a 12-phage peptide library, which has high affinity with diabetic ulcers in mice. Based on this, they prepared engineered placental MSCs, which overexpressed SGM on their surface and miR-146a in the cytoplasm using lentiviral vectors. A total of 24 diabetic BKS db/db mice were randomly and evenly divided into four groups. After creating full-thickness defects, treatment group 1 was coated with a soft silk fibroin patch (SFP) containing 100 μg SGM-Exo-miR-146a (SGM-Exo-miR-146a@SFP), treatment group 2 was only coated with 100 μg SGM-Exo-miR-146a, treatment group 3 was only coated with SFP, and the control group was untreated. The hemostasis speed of the three treatment groups was significantly faster than that of the control group, indicating that SGM-Exo-miR-146a, SFP, or SGM-Exo-miR-146a@SFP could all accelerate the wound healing. The wound areas were measured at days 3, 7, 14, and 21 after wounding, and mice treated with SGM-Exo-miR-146a, SFP, or SGM-Exo-miR-146a@SFP always healed faster than the control group. At day 21, only the SGM-Exo-miR-146a@SFP group healed completely, and the control group healed by only 60 %, indicating that miR-146a had additional healing promoting effects on the basis of SFP. Meanwhile, wound tissue from all groups was removed for skin function related tests, which showed that the SGM-Exo-miR-146a@SFP group induced the best healing effect. H&E staining revealed a well-arranged epithelium and regularly distributed keratinocytes, with complete hair follicles and sebaceous gland structure in the SGM-Exo-miR-146a@SFP group. Western blotting revealed an apparent decrease of IL-1R-associated kinase 1 and IL-6 levels, and an apparent increase in CD31 and VEGF levels. All these results suggested that miR-146a could promote the healing of diabetic ulcers by inhibiting the destructive effect of macrophages and promoting vascular growth.

#### 2.2.4 miR-182-5p

To explore the therapeutic potential of endothelial progenitor cell (EPC)-derived exosomes for diabetic ulcers, Li et al. established a full-thickness wound at the dorsum of diabetic mice [53], and the EPC-Exos greatly accelerated the speed of wound closure. To define the specific miRNA in EPC-Exos that participated in this process, the profiles of miRNAs were analyzed by sequencing, and one of the most upregulated was miR-182-5p. A dual-luciferase reporter assay indicated that miR-182-5p targets the mRNA encoding peroxisome proliferator-activated receptor gamma (PPARG) in keratinocytes. In *in vitro* proliferation or adhesion experiments in keratinocytes, an miR-182-5p antagomir suppressed their proliferation or adhesion. However, knockdown of *PPARG* counteracted the effects and recovered the proliferation or adhesion of keratinocytes, which confirmed that miR-182-5p promoted keratinocyte functionality by targeting *PPARG* mRNA. Eighteen diabetic mice induced by streptozotocin were randomly and evenly divided into three groups: The control group (saline), the miR-182-5p agomir group (10^8^ plaque forming units), and the agomir-NC group (10^8^ plaque forming units). The agomirs or saline were injected around the ulcer at day 0, 3 and 5. On the 7th day of treatment, only the ulcers of miR-182-5p agomir group were fully healed, whereas the ulcers of agomir-NC group shrank by 90 %, and those of the control group shrank by 80 %. The healing speed of the miR-182-5p agomir group was significantly faster than that of the agomir-NC and control groups. These results indicated that miR-182-5p promotes the healing of diabetic ulcers by targeting *PPARG* mRNA in skin keratinocytes.

### 2.3 Skin burns

Skin burns are commonly caused by heat, electricity, or chemicals, and the depth of the burn is classified as superficial, superficial partial-thickness, deep partial-thickness, and full-thickness. The severity of burns depends mainly on the size and depth of the defects, as well as the location, age, and presence of systemic diseases [54]. The managements of skin burns includes local coverage, skin grafting, fluid resuscitation, infection control, and nutrition [55]. Burn wounds consist of three distinguished zones of coagulation, stasis, and hyperemia [56]. The primary site of the injury is in the innermost zone, manifested as coagulative necrosis. The stasis zone consists of tissue damage and ischemia, but might still be salvageable. The outermost zone of hyperemia is characterized by redness and swelling caused by inflammatory cells (mainly macrophages) and cytokines (TNF-α and IL-1β) [57]. Among various burn models, the boiled water model has gained widespread use [58]. Boiled water at 80 °C (for deep partial-thickness burns) or 100 °C (for full-thickness burns) is applied for 8–10 s, which can create a circular burn area with a diameter of 2 cm.

#### 2.3.1 miR-181c

miRNA expression was compared between full-thickness scalded rats and normal rats using an miRNA array, and miR-181c expression was found to be deregulated in the scalded tissue [59]. Li et al. [60] speculated that miR-181c has a therapeutic effect on burns and reported its therapeutic effect on rats with full-thickness burns. UCMSCs were transfected with miR-181c using liposomes, and Exo-miR-181c were obtained from the culture supernatant. A total of 18 scalded rats were randomly and evenly divided into three groups: The control group (1 mL PBS), the Exo-miR-181c group (800 μg), and the UCMSC-Exo group (800 μg). All mice were euthanized at 24 h after treatment. Compared with the UCMSC-Exo group, administration of Exo-miR-181c dramatically decreased the total white blood cell count, and toll-like receptor 4, NF-κB/P65, and phosphorylated (p)-P65 protein levels were significantly decreased in the scalded tissue. Among serum inflammatory cytokines in the Exo-miR-181c group, the levels of TNF-α and IL-1β were significantly lower and level of IL-10 was higher than those of the other groups. Histological evaluation of cutaneous wounds showed that the number of neutrophils and macrophages in the Exo-miR-181c group were markedly lower than those in the UCMSC-Exo group. To explore the mechanism by which miR-181c suppressed macrophage-induced inflammation, toll-like receptor 4, NF-κB/P65, and p-P65 protein levels were assessed in macrophages and were observed to be significantly downregulated. These results suggested that miR-181c could reduce burn-associated inflammation by targeting the mRNA encoding toll-like receptor 4 in macrophages.

### 2.4 Hypertrophic scars

A hypertrophic scar (HTS) develops generally after a full-thickness skin defect or severe burn, and is a fibro-proliferative disorder manifesting as transdifferentiation of fibroblasts into myofibroblasts, which in turn synthesize excessive alpha smooth muscle actin (α-SMA), collagen-I, and collagen-III through the TGF-β/Smad3 signaling pathway [61]. HTS needs to be differentiated from keloids. HTS is limited to the original wound, whereas keloids extend beyond the original wound. HTS usually appears 1–2 months after injury and regresses after a 6-month rapid growth period [62]. Conservative therapies (compression therapy, gel sheets, scar massage, corticosteroids, and lasers) can reduce the HTS volume, and suppress pain and itching [63]. Many animal models, such as mice, rats, rabbits, and pigs have been used to study the formation of HTS; however, there is no consensus on which model is closest to humans [64]. Mice are the most widely used species, and skin burns and excisional full-thickness wounds are the widely used models, which is partly because of the availability of mouse-derived detection antibodies and their low cost.

#### 2.4.1 miR-29a

Yuan et al. [65] found that the expression levels of miR-29a were significantly downregulated in human burn scars and hypertrophic scar fibroblasts compared with those in normal tissue and fibroblasts, respectively. Therefore, it was speculated that miR-29a could accelerate healing and reduce burn scars. miR-29a mimics were transfected into human ADSCs using liposomes, and Exo-miR-29a was obtained from the culture supernatant. Eighteen mice with full-thickness burns were randomly and evenly divided into three groups: Treatment group 1 and treatment group 2 were subcutaneously injected with 200 μg Exo-miR-29a and 200 μg Exo-miR-29a+30 mg/kg TGF-β agonist, respectively, and the control group received PBS. All mice were treated every 3 days and euthanized at 15 days after scalding. From the gross observation of wound healing and scar formation, Exo-miR-29a showed an obvious effect on the scalded skin, and the TGF-β agonist reversed this effect. During histological examination, a thick dermis, disordered granulation tissue, dense collagen fibers, and obvious infiltration of inflammatory cells were observed in the control group and the Exo-miR-29a/TGF-β agonist group. Exo-miR-29a markedly reduced the aforementioned situation in the wound. Exo-miR-29a inhibited the activation of TGF-β/Smad3 signaling, thereby decreasing the expression of α-SMA, collagen-I, and collagen-III in scar tissues, while the TGF-β agonist reversed this inhibitory effect. These results indicated that miR-29a inhibits HTS formation by blocking the TGF-β/Smad3 pathway in fibroblasts.

#### 2.4.2 miR-192-5p

miR-192-5p was reported to exert an anti-fibrotic effect in renal fibrosis [66]; therefore, Li et al. [67] cocultured HTS-derived fibroblasts with human ADSC-Exos for 24 h. The proliferation and migration of fibroblasts significantly slowed down, while the content of α-SMA, collagen-I, collagen-III, and IL-17 receptor α chain (IL-17Rα) decreased. After HTS-derived fibroblasts were transfected with miR-192-5p mimics or inhibitors using liposomes, the expression of IL-17Rα decreased after mimic transfection and increased after inhibitor transfection. This result indicated that miR-192-5p targeted the mRNA encoding IL-17Rα in fibroblasts, which was then verified using a dual-luciferase reporter assay. Twelve mice with full-thickness defects on their dorsal skin were randomly and evenly divided into a control group (PBS) and an ADSC-Exo group. ADSC-Exos significantly accelerated wound healing compared with that in the control group. In the HTS samples harvested on day 14, the expression of α-SMA, collagen-I, and collagen-III in the ADSC-Exo group was significantly lower than that in the control group, and the ADSC-Exo group showed less collagen deposition, and a thinner, orderly arranged structure. Subsequently, knockdown of *IL1*7RA decreased the expression of α-SMA, collagen-I, and collagen-III in HTS-derived fibroblasts, and accelerated the healing of full-thickness defects. These results clarified that the anti-fibrotic effect of miR-192-5p was realized by targeting IL-17Rα expression in fibroblasts.

### 2.5 Psoriasis

Psoriasis is a chronic, autoimmune cell-mediated inflammatory skin disease characterized by skin erythema covered with silver scales, most commonly found in the elbows, knees, scalp, and lower back. Psoriasis is mainly associated with psychological, metabolic, arthritis, and cardiovascular symptoms, with pathological characteristics of imbalanced epidermal cell proliferation and differentiation, and increased angiogenesis and infiltration of immune cells [68]. Increased expression of IL-23 (secreted by dendritic cells) and IL-17 (secreted by Th17 cells) can be found in the serum from patients with psoriasis [69]. In skin lesions from patients with psoriasis, levels of phosphorylated signal transducer and activator of transcription 3 (STAT3) were higher than those in normal skin [70]. Currently, treatments for psoriasis include hormone ointment for external use, oral medication, phototherapy, biological agents, and traditional Chinese medicines [71]. For psoriasis models, it is usual to shave the back area of the mouse at 2 × 3 cm first, and then apply 50 mg of 5 % Imiquimod for 3 consecutive days [72]. The skin will gradually develop psoriasis-like inflammatory reactions, such as scales, erythema, and epidermal thickening. The Psoriasis Area and Severity Index is used to score for erythema, scaling, and thickness of the skin lesions, with 0–4 points representing symptoms ranging from healthy skin to very severe skin conditions [73].

#### 2.5.1 miR-124-3p

Abnormal elevation of IL-17A expression is often found in various autoimmune diseases; consequently, Liu et al. [74] identified miR-124-3p as one of the differentially expressed miRNAs in IL-17A-treated human keratinocytes, and the dual-luciferase reporter assay showed that *STAT3* mRNA was targeted by miR-124-3p. Therefore, the authors treated Imiquimod-induced mice with miR-124-3p. Human keratinocytes were cultured *in vitro*, exosomes were isolated, and miR-124-3p was transfected by electroporation to obtain Exo-miR-124-3p. A total of 20 psoriatic mice were randomly and evenly divided into three groups, 3 μg miR-124-3p mimic or miR-NC were packaged in liposomes or 50 μg Exo-miR-124-3p were injected into the back skin at days 0, 1 and 3. The Psoriasis Area and Severity Index scores showed that the miR-124-3p mimic group and Exo-miR-124-3p group had relieved skin inflammation and reduced skin thickness (both by an average 2.1 points) compared with those in the miR-NC group (average 3.3 points). The epidermal thickness, proliferating cells, and the oxidative stress marker malondialdehyde all decreased significantly in the miR-124-3p mimic and Exo-miR-124-3p groups, but not in the miR-NC group. The above results showed that miR-124-3p could inhibit psoriasis-like skin inflammation by targeting *STAT3* mRNA in keratinocytes.

### 2.6 Systemic sclerosis

Systemic sclerosis (SSc) is a chronic autoimmune connective tissue disorder, characterized by skin fibrosis, inflammation, and lung vasculopathy. The disease presents with arthritis, gastrointestinal diseases, myositis, and Raynaud's phenomenon in the early stage, and fatal pulmonary or cardiac complications in the late stages [75]. Disease progression primarily involves endotheliocyte activation, and the recruitment of macrophages and T cells, followed by abnormal differentiation of fibroblasts and excessive deposition of the extracellular matrix [76]. Pathological features of SSc include massive invasion of myofibroblasts, accumulation of various proteins secreted by fibroblasts, and upregulation of profibrotic TGF-β [77], which stiffens the skin and organs, and decreases their elasticity. Currently, there is a lack of specific therapies, thus current clinical management comprises symptomatic treatment combined with systemic immunosuppression [78]. There are multiple murine models that use subcutaneous fibrosis-inducing agents (i.e., bleomycin) or prooxidative agents (i.e., hydroxyl radicals and hypochlorous acid [HClO]) to simulate SSc [79]. The bleomycin-induced model is the most appropriate model, which simultaneously possesses the three characteristics of skin fibrosis, inflammation, and lung vasculopathy; however, the fibrosis resolves over time with discontinuation of induction [80]. The mice induced by hydroxyl radicals or HClO have stable characteristics of skin fibrosis and lung vasculopathy; however, the systemic inflammation is mild [81].

#### 2.6.1 miR-29a-3p

miR-29a was reported to target the mRNA encoding TGF-β-activated kinase 1 binding protein 1 in fibroblasts, thereby disrupting the production of anti-apoptotic factors B-cell lymphoma-2 and B-cell lymphoma-XL [82]. Rozier et al. [83] divided 16 HClO-induced SSc mice randomly and evenly in 2 groups: The control group (100 μL PBS) and the ADSC-Exo group (4 × 10^7^ particles). ADSC-Exos were injected intravenously, which reversed the disease course and reduced skin thickness in 6 weeks, with reduced fibrotic remodeling and inflammatory markers in serum. Subsequently, another 16 SSc mice were randomly and evenly divided into two groups: The miR-29a-3p antagomir group and the antagomir-NC group. The miR-29a-3p antagomir did not reverse the disease course and reduce the skin thickness in 6 weeks, meanwhile excessive collagen deposition was still observed in the skin lesions. DNA demethylase Tet methylcytosine dioxygenase 1, DNA methyltransferase 3A, platelet-derived growth factor receptor B, B-cell lymphoma-2, and B-cell lymphoma-XL were all expressed at high levels in skin lesions of the miR-29a-3p antagomir group, but were significantly decreased in the antagomir-NC group. These results suggested that miR-29a-3p mediates the therapeutic effect of ADSC-Exos in the SSc model by modulating the expression of multiple genes in fibroblasts.

#### 2.6.2 miR-196b-5p

To assess the effect of MSC-Exos on SSc, Baral et al. [84] used the bleomycin-induced mouse model. Bleomycin-enhanced dermal thickness, dermal fibrosis, increased skin collagen, and α-SMA^+^ myofibroblasts were all significantly suppressed by injection of mouse BMSC-Exos, suggesting their potential to suppress skin fibrosis. miRNA analysis was performed to compare BMSC-Exo and dermal fibroblast-Exos using micro-arrays, which revealed that miR-196b-5p was accumulated at the highest concentration in BMSC-Exos, and dual-luciferase reporter assays showed that the target was the mRNA encoding collagen type 1 alpha 2 (COL1A2). Therefore, miR-196b-5p was speculated to be a key factor in the anti-fibrotic function of BMSC-Exos, and amiR-196b-5p mimic was transfected to test the anti-fibrosis effect in TGF-β-treated fibroblasts. Overexpression of miR-196b-5p significantly suppressed TGF-β-induced upregulation of *COL1A2* expression. These results suggested that miR-196b-5p could treat SSc by targeting *COL1A2* mRNA in fibroblasts.

#### 2.6.3 miR-214

In patients with SSc, miR-214 was found to be downregulated in plasma [85]. Xie et al. [86] compared the lesioned skin of 66 patients with SSc with the skin of 42 healthy volunteers, and upregulated IL-33 and downregulated miR-214 expression was found in the patients with SSc. Similar conclusions were obtained when comparing bleomycin-induced mice with normal mice. An miR-214 mimic was transfected using liposomes into TGF-β-treated fibroblasts, and western blotting revealed decreased IL-33 levels. Combined with dual-luciferase reporter assays, the authors concluded that miR-214 targets *IL-*33 mRNA in TGF-β-treated fibroblasts. Subsequently, BMSC-Exos were extracted to treat bleomycin-induced SSc mice for three weeks. The control group received PBS. Compared with that in the control group, miR-214 was expressed at a high level and IL-33 at a low level in the lesioned skin of the BMSC-Exo group. The BMSC-Exo group also showed decreased dermis thickness, α-SMA levels, and collagen fiber accumulation in the lesions. Overall, BMSC-Exo-delivered miR-214 could treat SSc by targeting the *IL-*33 mRNA in fibroblasts.

### 2.7 Atopic dermatitis

Atopic dermatitis (AD) is a chronic inflammatory skin ailment that might have autoimmune mechanisms, characterized by eczema, intense pruritus, and dry skin [87]. Treatments for AD include reducing inflammation, maintaining the barrier function of the skin, alleviating pruritus, and avoiding superinfections caused by stratum corneum degradation [88]. For AD models, rodent skin was shaved and exposed to allergens or microorganisms, such as house dust mites, 2,4,6-trinitro-1-chrolobenzene, and oxazolone [89]. Epidermal sensitization elicits the production of antigen-specific IgE and a Th2 immune reaction, which change the skin barrier to induce infiltration of inflammatory cells and thickening of the epidermis.

#### 2.7.1 miR-147a

Considering the anti-inflammatory effect of miR-147a in keratinocytes [90] and endotheliocytes [91], miR-147a might be a potential therapeutic drug to treat AD. For AD mice induced by *Dermatophagoides farinae* extract and 2,4-dinitrochlorobenzene, Shi et al. [92] found that miR-147a levels decreased markedly in the serum and skin lesions, meanwhile thymic stromal lymphopoietin (TSLP, a cytokine involved in the angiogenic phenotype and AD pathogenesis) and VEGFA (a key regulator of angiogenesis) increased markedly. These observations revealed that miR-147a correlated negatively with the expression levels of VEGFA and TSLP in AD lesions. Subsequently, Exo-miR-147a was produced by transfecting an miR-147a mimic into ADSCs using liposomes, which then attenuated the TNF-α/interferon-γ-induced inflammatory response and apoptosis of human epidermal keratinocytes, and suppressed the angiogenesis of endotheliocytes. Using dual-luciferase reporter assays, miR-147a was found to target the myocyte enhancer factor 2A-TSLP axis in keratinocytes and target *VEGFA* mRNA in endotheliocytes. These results suggested that miR-147a could treat AD by inhibiting both pathological angiogenesis and inflammatory injury synchronously.

### 2.8 Skin aging

Aging is a biologically natural process, and the hallmarks of skin aging are a decrease in the thickness of the dermis and increased accumulation of subcutaneous fat [93]. The reasons for skin aging are divided into intrinsic and extrinsic causes. The intrinsic causes comprise physiological age, excessive smoking, alcoholism, and the intake of carbohydrates, characterized by apoptosis of fibroblasts via the TGF-β-activated kinase 1 binding protein 1 (TAK1) pathway [4]. The extrinsic factors usually refer to skin aging caused by ultraviolet radiation, air pollution, chronic diseases, and bad lifestyles [94]. Excessive ultraviolet radiation can induce skin wrinkle formation, epidermal thickening, and collagen fiber loss via the MAPK/activator protein-1 (AP-1) pathway, which is termed photoaging [95]. The classic skin photoaging model was prepared using ultraviolet B sunlamps with a peak radiation at 310 nm [96,97]. d-galactose is able to trigger aging-like symptoms in animal models, and the organs involved include the brain, heart, liver, kidney, lung, and skin [98].

#### 2.8.1 miR-767 inhibitor

When screening for aging-related miRNAs from senile skin, Li et al. [99] found that the expression of miR-767 decreased in d-galactose-treated fibroblasts and increased in d-galactose-treated endotheliocytes. Moreover, exosomes derived from d-galactose-treated endotheliocytes could accelerate the aging of fibroblasts. Dual-luciferase reporter assays revealed that miR-767 targeted the mRNA encoding TAK1 in fibroblasts. Transfection with an miR-767 mimic or inhibitor promoted or reversed the aging of fibroblasts. Four C57BL/6 mice were randomly and evenly divided into two groups: The control group (back injection with 70 μL of saline) and the miR-767 mimic group (5 μg/μL). Compared with the control group, the epidermis of the miR-767 mimic group was significantly thinner, and the collagen layer was reduced, suggesting that miR-767 might induce skin aging. These results revealed that the inhibition of miR-767 might reverse the aging of skin fibroblasts.

#### 2.8.2 miR-1246

Both ultraviolet A and B radiation were reported to markedly downregulate the expression of miR-1246 in skin fibroblasts [100]. Consequently, Gao et al. [101] deduced that miR-1246 plays a core role in the therapeutic effect of MSC-Exos on skin photoaging. Exo-miR-1246 and Exo-miR-NC were obtained from the culture supernatants of each group. Results of *in vitro* experiments showed that Exo-miR-1246 blocked the AP-1 signaling pathway and increased collagen-I secretion by activating the TGF-β/Smad pathway in fibroblasts. Twenty-four mice were randomly and evenly divided into four groups. Human ADSCs were transfected with lentivirus expressing the miR-1246 mimic (Exo-miR-1246 group) and miR-NC (Exo-miR-NC group), respectively, the remaining mice were the irradiation only group and the non-irradiated group. Exosomes were injected through tail vein at a dose of 5 × 10^9^ particles. After 8 weeks of irradiation, all mice exhibited significant photoaging, characterized by coarse wrinkles, sagging, and dryness. Treatment with exosomes improved the symptoms to different degrees. In particular, Exo-miR-1246 reversed the skin condition to resemble that of the non-irradiated group. After 4 weeks, the epidermal thickening and collagen reduction were reversed by Exo-miR-1246 treatment, with large deposits of collagen fibers in the dermis. The above results suggested that miR-1246 could reduce skin wrinkle formation, epidermal thickening, and collagen fiber loss.

### 2.9 Hair loss

Most people usually have about 100,000 scalp hairs, and 100–150 telogen hairs are shed every day. In healthy conditions, the total number and density of hair remain stable. Hair growth occurs in four continuous phases (anagen, catagen, telogen, and exogen), representing the growth, regression, rest, and shedding of hair, respectively. Each hair follicle will cycle independently for 10–30 cycles [102]. Hair loss is the most visible head feature and is an important external manifestation of aging caused by factors such as nutritional deficiencies, poor sleep, stress, androgenic alopecia, and inflammation. Androgenic alopecia is the most common reason for hair loss, in which high concentrations of dihydrotestosterone (DHT) in the scalp cause progressive shrinkage of dermal papilla cells (DPCs) [103]. Many therapies improve hair loss, including increased blood flow, direct stimulation of the hair follicle, growth factors, and platelet-rich plasma [104]. Methods to increase blood flow on the scalp include scalp massage and Minoxidil, and methods to stimulate hair follicles include prostaglandins and lasers. For an animal model of hair loss, DHT can induce regression and morphological change of DPCs in male C57BL/6 mice in a dose-dependent manner [105]; the skin remained gray after 7 days of anagen, and then turned white again with the apoptosis of melanocytes [106].

#### 2.9.1 miR-122-5p

Liang et al. [107] found that ADSC-Exos could reverse the inhibitory effects of DHT on DPCs and downregulated the TGF-β/Smad3 axis. High-throughput sequencing identified 225 co-expressed miRNAs in ADSC-Exos and DPCs. Among them, miR-122-5p had the highest expression intensity. Using dual-luciferase reporter assays, miR-122-5p was identified to target the mRNA encoding Smad3 in DPCs. Therefore, miR-122-5p might counteract the inhibitory effect of DHT on hair follicles. Human ADSCs were transfected with lentivirus loaded with an miRNA-122-5p mimic or an miRNA-122-5p inhibitor, and Exo-miRNA-122-5p or its inhibitor were harvested from the culture media. Thirty C57BL/6 mice were randomly and evenly divided into five groups after back shaving: The control group (200 μL PBS), the DHT group (10^−5^ mol/L), the DHT + Exo-miR-122-5p group (10^−5^ mol/L+50 μg/mL), the DHT + Minoxidil group (10^−5^ mol/L+200 μL), and DHT + Exo-miR-122-5p inhibitor group (10^−5^ mol/L+50 μg/mL). The drugs were injected every day at six points on the dorsal skin. By day 11, the skin of the DHT + Minoxidil group and DHT + Exo-miR-122-5p group gradually became gray, indicating that the DPCs had entered the anagen phase, while the skin of DHT group was still pink. On day 15, the DHT + Exo-miR-122-5p and DHT + Minoxidil groups both showed a larger area of hair coverage, thicker dermis, and more hair bulbs than the other groups, indicating the antagonistic effect of miR-122-5p on DHT. The DHT + Exo-miR-122-5p inhibitor group showed less hair coverage, smaller DPCs, and thinner skin compared with the control group. These results suggested that miR-122-5p can counteract androgenic alopecia by inhibition of the TGF-β/Smad3 axis in DPCs.

## 3 Conclusions

**Fig. 1:**
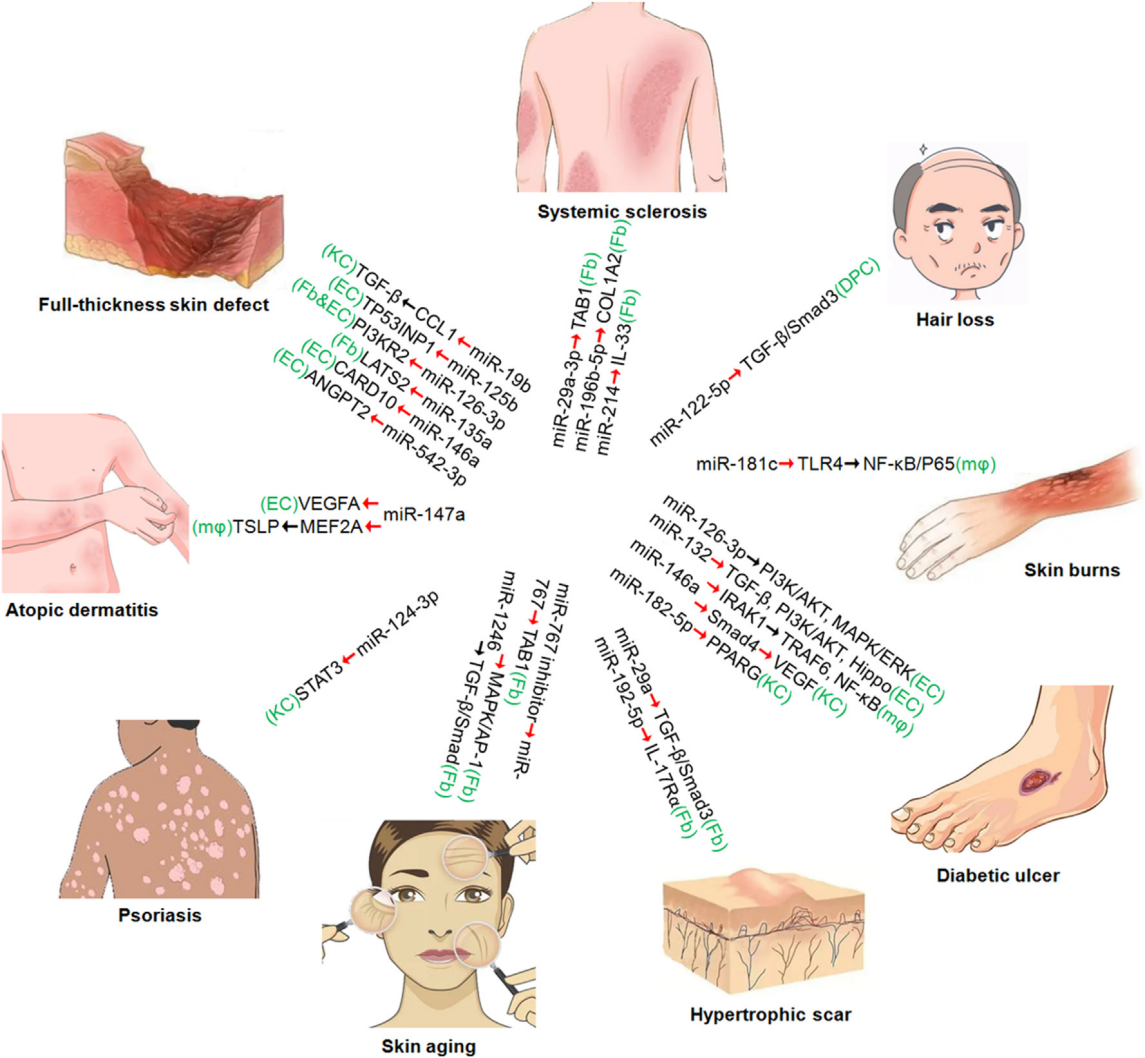
Twenty-one exosomal microRNA-based therapies for nine skin diseases. The red arrow in the figure represents targeted binding, the black arrow represents activation, and the green brackets represent the type of effector cells. miR, microRNA; Fb, fibroblasts; DPC, dermal papilla cell; mφ, macrophage; EC, endothelial cell; KC, keratinocyte.

The regulation of miRNA expression is a novel and promising therapy to solve multiple refractory skin problems. The exogenous delivery of anti-inflammatory miRNA mimics/agomirs or inhibitors/antagomirs are beneficial to inhibit proinflammatory mediators, thereby alleviating multiple skin problems (Fig. 1). Multiple gene modification methods can arbitrarily insert miRNA-related genes into exosomes to become drugs (Table 1). It is crucial to fully evaluate the best indications, safety issues, potential off-target effects, delivery methods, and translational hurdles of miRNA therapies. Most studies feature small animal samples without evidence of replication; therefore, these therapies need to be effectively addressed using standardized models and measures. Furthermore, comparing miRNA therapies with existing standard treatments will be invaluable in the future.

**Table 1:** Targeted uses of microRNA drug therapy.

Skin diseases	miRNA Drug	Target cell	Target mRNA	Signal pathway	Ref
Full-thickness skin defect	miR-19b	KC	CCL1	TGF-β↓	32
	miR-125b	EC	TP53INP1		33
	miR-126-3p	Fb&EC	PI3KR2		30
	miR-135a	Fb	LATS2		34
	miR-146a	EC	CARD10		36
	miR-542-3p	EC	ANGPT2		38
Diabetic ulcer	miR-126-3p	EC		PI3K/AKT↑, MAPK/ERK↑	47
	miR-132	EC		TGF-β↓, PI3K/AKT↓, Hippo↓	49
	miR-146a	mφ	IRAK1	TRAF6↓, NF-κB↓	52
	miR-146a	KC	Smad4	VEGF↑	52
	miR-182-5p	KC	PPARG		53
Skin burns	miR-181c	mφ	TLR4	NF-κB/P65↓	60
Hypertrophic scar	miR-29a	Fb		TGF-β/Smad3↓	65
	miR-192-5p	Fb	IL-17Rα		67
Psoriasis	miR-124-3p	KC	STAT3		74
Systemic sclerosis	miR-29a-3p	Fb	TAB1		83
	miR-196b-5p	Fb	COL1A2		84
	miR-214	Fb	IL-33		86
Atopic dermatitis	miR-147a	EC	VEGFA		92
	miR-147a	mφ	MEF2A	TSLP↓	92
Skin aging	miR-767	Fb	TAB1		99
	miR-1246	Fb		MAPK/AP-1↓, TGF-β/Smad↑	101
Hair loss	miR-122-5p	DPC		TGF-β/Smad3↓	107

Note: Fb, fibroblasts; DPC, dermal papilla cell; mφ, macrophage; EC, endothelial cell; KC, keratinocyte.

## Author contributions

Conception and design: Chen Ji-bing, Feng Bing-zheng and Xu Zhi-ran. Drafting of the manuscript: Chen Ji-bing, Liang Wei-ping and Yang Yu-wei.

## Funding

This research was supported by a grant from the Chinese Medicine Service System and Capacity Building (Key Project with Chinese Medicine Characteristics and Advantages, Ruikang Hospital, 2023).

## Data availability statement

Not applicable.

## Declaration of competing interest

No conflicts of interest regarding any of the authors of this manuscript.
